# Assessment of dietary intake and mineral status in pregnant women

**DOI:** 10.1007/s00404-018-4744-2

**Published:** 2018-03-14

**Authors:** Rafał Kocyłowski, Iwona Lewicka, Mariusz Grzesiak, Zuzanna Gaj, Anna Sobańska, Joanna Poznaniak, Constantin von Kaisenberg, Joanna Suliburska

**Affiliations:** 1 Department of Perinatology and Gynecology, Polish Mother’s Memorial Hospital Research Institute, Lodz, Poland; 2 PreMediCare New Med Medical Centre, Poznan, Poland; 3 Institute of Human Nutrition and Dietetics, Poznan University of Life Sciences, ul. Wojska Polskiego 31, 60-624 Poznan, Poland; 4 Department of Gynecology and Obstetrics, Polish Mother’s Memorial Hospital Research Institute, Lodz, Poland; 5 Scientific Laboratory of the Center of Medical Laboratory Diagnostics and Screening, Polish Mother’s Memorial Hospital-Research Institute, Lodz, Poland; 6 Department of Obstetrics, Gynecology and Reproductive Medicine, Hannover Medical School, Hannover, Germany

**Keywords:** Pregnancy, Dietary habits, Minerals, Folic acid, Vitamin D

## Abstract

**Purpose:**

To evaluate the dietary intake of pregnant women and their nutritional status of Ca, Mg, Fe, Zn, and Cu, as the nutritional status of pregnant women is an important factor for the proper progression of a pregnancy and the development and health of the foetus.

**Methods:**

The study was conducted on 108 pregnant women ages 18–42, at 6–32 weeks of gestation. We used a questionnaire and a 24-h recall nutrition interview. Hair samples were taken for testing and the level of each mineral was assessed using atomic absorption spectrometry. The results were analysed using the Dietetyk and Statistica 10 software.

**Results:**

Low levels of Fe, Zn, Ca, Mg, vitamin D, and folic acid intake were seen in the pregnant women, with the use of dietary supplements significantly increasing their intake of Fe, Zn, and folic acid. The concentration of zinc and magnesium in the women’s hair was shown to be affected by their age and, in the case of magnesium, by the week of pregnancy.

**Conclusions:**

It was observed that the diet of pregnant women is characterised by low levels of Fe, Zn, Ca, Mg, vitamin D, and folic acid. Dietary supplementation with vitamins and minerals significantly increases the daily Fe and folic acid intake in pregnant women. The concentration of Zn and Mg in hair depends on the age of pregnant women and Mg level in the hair of women decreases during pregnancy.

## Introduction

The dietary habits of pregnant women are important for the proper progression of pregnancy and the development and health of the foetus [1–6]. During pregnancy, diets should be balanced in terms of macronutrients and micronutrients. The daily energy requirements for healthy women of normal weight and who have a moderately active lifestyle, increase during pregnancy and are based on the trimester of the foetus [7]. Recommendations regarding the calorific value of pregnant women’s diets aim to prevent the development of obesity [8]. As the pregnancy progresses, the woman’s need for protein increases, peaking in the third trimester. The appropriate intake of protein in the diet supports the protein biosynthesis needed to supply the needs of maternal tissues, the placenta, and the growing foetus [8]. The total fat intake, especially in the first trimester of pregnancy, should not increase significantly [8].

Studies conducted so far indicate the particular importance of micronutrients during pregnancy [9]. Iron deficiency in pregnant women is a cause of anaemia and predisposes women to urinary tract infections. Iron deficiency anaemia in pregnant women significantly correlates with a low birth weight of newborns and a higher risk of premature birth [10, 11]. The demand for iron during pregnancy increases significantly, especially in the third trimester [10]. According to the current recommendations, the diet should deliver 27 mg of iron per day to pregnant women [12]. Calcium deficiencies in the diet of pregnant women lead to decreased bone density in both the mother and the child. Inadequate calcium intake also correlates with hypertension in a pregnant woman, and thus increases the risks of premature birth and newborn mortality [13, 14]. According to the current recommendations, the daily calcium requirement for pregnant women is 1200–2000 mg, and the maximum tolerated dose is set at 2500 mg [15], although there is limited evidence for calcium supplementation in preeclampsia prevention [16]. During pregnancy, the body’s daily demand for zinc increases to 11–12 mg. Studies have shown a positive correlation between inadequate zinc intake during pregnancy and increased risk of premature birth, low birth weight, and foetal developmental defects. Zinc deficiency in pregnant women is also associated with a higher risk of hypertension, eclampsia, infection, and prolonged labour [17–19]. Copper deficiency during pregnancy can result in anaemia and neutropenia, increase susceptibility to infections, and promote metabolic disturbances in glucose and cholesterol. It has also been suggested that the concentration of copper in the pregnant woman’s body can have an effect on the anthropometric parameters of the foetus [20–22]. The recommended daily intake of copper for pregnant women is 1 mg [23]. Studies conducted so far indicate that there is a significant increase in the amount of magnesium needed by pregnant women, which can be as high as twice the pre-pregnancy requirement [24]. Depending on the extent of magnesium deficiency, and given its specific risks in pregnant women, magnesium supplementation is suggested, with a dosage in the range of 200–1000 mg [25]. Magnesium deficiency during pregnancy can result in hypertension, pre-eclampsia, painful muscle contraction, and migraines. Studies show the association of magnesium deficiency with an increased risk of premature birth, gestational diabetes, and foetal growth disorders [25].

The appropriate intake of folic acid, and its concentration in pregnant women’s bodies, significantly affects the normal development of the foetus. Folic acid plays a key role in the synthesis of nucleic acids and in the homocysteine and amino acid metabolism. Folic acid supplementation at 0.4 mg a day is widely recommended for potentially childbearing women [8]. The proper intake of folic acid at least 3 months before conception and during 2–3 months of pregnancy reduces the risk of neural tube defects and congenital heart defects, and promotes the normal development of the placenta [8]. Recent studies advise that all pregnant women take vitamin D_3_ supplementation at a dose of 600 IU per day [26, 27]. It is thought that the improper intake of vitamin D_3_ during pregnancy increases the risk of premature birth and the development of pre-eclampsia. However, the role of vitamin D in pre-eclampsia (PE) is not entirely clear, and the independent effect of vitamin D supplementation in preventing PE has not been confirmed [28]. Study results indicate the association of vitamin D_3_ deficiency with low birth weight, abnormal bone development, increased risks of respiratory tract infection, and early childhood allergy [26, 27]. Table 1 presents the current recommendations for the intake of these macronutrients and micronutrients during pregnancy.

**Table 1:** Recommended intakes of energy and selected macro and micronutrients in pregnant and non-pregnant women [1, 8, 22]

Nutrients	Non-pregnant women^a^	Pregnant women		
		I trimester	II trimester	III trimester
Energy (kcal/day)	2000–2200	+ 70^a^	+ 260^a^	+ 500^a^
Protein (g/day)	41–72	+ 1^a^	+ 8^a^	+ 26^a^
Fats (g/day)	67–73	–	+ 8–14^b^	+ 11–18^b^
Carbohydrates (%E^c^)	50–70%	50–70%		
Calcium (mg/day)	1000	1200	1500–2000	
Magnesium (mg/day)	310–320	375		
Iron (mg/day)	18	27		
Zinc (mg/day)	8	11–12		
Copper (mg/day)	0.9	1		
Folic acid (µg/day)	400	0.6		
Vitamin D (μg/day)	5	10–20		

^a^Recommended intakes for women in childbearing age, with normal weight and average physical activity

^b^In relation to individual nutritional demand before pregnancy

^c^%E =  % of total energy intake

## Materials and methods

The research was carried out with the approval of the Local Ethical Committee at the Poznan University of Medical Sciences (Approval No. 297/17) and the Research Ethical Committee of the Polish Mother’s Memorial Hospital Research Institute (Approval No. 50/2016). The study was supported by the statutory research funding from Institute of Human Nutrition and Dietetics at Poznan University of Life Sciences (No. 508-786-00) and partially funded by the Polish Ministry of Science and Higher Education for Polish Mother’s Memorial Hospital Research Institute in Lodz (Grant No. 2016/I/18-GW).

The study involved 108 European low-risk pregnant women from Greater Poland with ages ranging from 18 to 42. It was noticed that 48% women were in their first pregnancy. Average parity of women was 1.7 ± 1.1 and average gravidity 1.8 ± 1.2.

The study used a validated questionnaire which we devised, and a 24-h recall nutrition interview. The interview results were analysed using the Dietetyk software. Hairs taken from six places on the occipital part of the head were used for the biochemical analysis. The hair was removed by cutting the strands close to the skin. Hair sections of 2 cm in length were used for the analysis, in which calcium, magnesium, iron, copper, and zinc levels were determined by atomic absorption spectrometry using an AAS-3 spectrophotometer (Carl Zeiss, Germany). The accuracy of the assay was 91, 98, 94, 94, 102% for calcium, magnesium, zinc, iron and copper, respectively, as verified by certified reference materials (Human Hair NCS DC73347a, LGC).

Statistical analysis of the results was performed using Statistica 10 software, employing the nonparametric test, the Mann–Whitney test, the Chi^2^ test, the Kruskal–Wallis ANOVA, and the Spearman correlation coefficient. Significance was taken to be at the *p* < 0.05 level.

## Results

The study population consisted of 108 pregnant women of average age 31.37 ± 4.87. During the study, the women were in week 17.7 ± 5.3 of pregnancy on average (ranging from week 6 to week 32). The detailed characteristics of the women are set out in Table 2.

**Table 2:** Characteristics of women participating in the study

Parameters	Value	
Total number of women	108	
Average age (years)	31.4 ± 4.9	
	< 35	≥ 35
	75 women (69%)	33 women (31%)
Week of gestation	17.7 ± 5.3	
	≤ 19	≥ 20
	54 (50%)	54 (50%)
Average body weight before pregnancy (kg)	60.6 ± 12.6	
Status of nutrition before pregnancy (%)^a^	Underweight (BMI < 18.5 kg/m^2^)	18
	Normal weight (BMI 18.5–24.9 kg/m^2^)	71
	Overweight (BMI 25–29.9 kg/m^2^)	12
	Obese (BMI ≥ 30 kg/m^2^)	7
Average body weight during pregnancy (kg)	64.8 ± 13.0	

^a^Status of nutrition before pregnancy was determined based on the BMI value, according to the WHO classification

Table 3 shows the characteristics of the pregnant women’s diets in terms of the daily intake of macronutrients. The average energy value of the diet of the pregnant women was below the recommended amount [7]. Likewise, the intake of carbohydrates was insufficient [8]. The intake of protein and fat in the diet slightly exceeded the recommendations [8]. Surprisingly, it was found that the lowest fat intake was found among obese women, while the highest intake was among underweight women. Similarly, in the case of energy consumption, the lowest intake was in the case of the obese pregnant women, and the highest intake was among the underweight women.

**Table 3:** Daily intake of macronutrients in the diet of pregnant women

Macronutrients	Mean ± SD (%RDA)
Energy (kcal/day)	1964.9 ± 345.7 (87)
Protein (g/day)	80.6 ± 18.8 (103)
Carbohydrates (g/day)	269.8 ± 58.7 (82)
Fats (g/day)	72.6 ± 20.9 (105)
Cholesterol (mg/day)	272.4 ± 132.1 (90.8)
Fibre (g/day)	25.2 ± 10.4 (100.8)

*SD* standard deviation, *RDA* recommended dietary allowance

The results showed that almost half of the pregnant women did not supplement their diet with vitamins or minerals (Table 4). Of those who did supplement, more than 90% took folic acid, with a much smaller proportion taking iron.

**Table 4:** Percentage of pregnant women using dietary supplementation

Parameter	Percentage of women (%)
Use of supplements during pregnancy (*n* = 58)	53.7
Categories of supplements	
Composite vitamin and mineral supplements	17.6
Vitamin D preparation	15.7
Magnesium preparation	5.6
Folic acid preparation	93
Iron preparation	16

Table 5 shows the extent to which the micronutrient intake recommendations were met, counting both diet and the diet with supplements. The average amount of iron in the diet was 30% of the recommended amount. Supplementation resulted in a significant increase in daily iron intake. Similarly, the average values of calcium and magnesium intake in the diet were not consistent with the recommendations. Supplementation increased the intake of calcium and magnesium, but the supply still remained below the recommendation. The average zinc intake in the diet was nearly 90% of the recommended amount. Supplementation with zinc increased the intake of this nutrient to above the recommended amount. The average intake of copper in the diet slightly exceeded the recommendation, with supplementation further increasing its daily intake. The average vitamin D intake was significantly lower than the recommendation. Supplementation with vitamin D led to an increase in its intake, and the recommended daily allowance was reached. Similarly, the average intake of folic acid in the diet was not in line with the recommendations; however, dietary supplementation with folic acid resulted in a significant increase in its daily intake, even exceeding the recommended level.

**Table 5:** Average daily intake of micronutrients with diet and supplements (*n* = 108)

Micronutrients	Intake with diet	Intake with diet and supplements
	Mean ± SD (%RDA)	Mean ± SD (%RDA)
Iron (mg/day)	10.5 ± 2.5 (39)	30.1 ± 19.1 (112)
Zinc (mg/day)	10.5 ± 2.9 (91)	12.7 ± 3.2 (116)
Magnesium (mg/day)	271.5 ± 77.3 (75)	326.5 ± 70.0 (91)
Calcium (mg/day)	838.5 ± 360.3 (84)	885.3 ± 357.2 (89)
Copper (mg/day)	1.2 ± 0.3 (123)	1.6 ± 0.5 (157)
Vitamin D (µg/day)	1.8 ± 3.3 (12)	11.2 ± 1.2 (75)
Folic acid (µg/day)	253.8 ± 104.9 (42)	746.4 ± 275.8 (124)

*RDA* recommended dietary allowance

Tables 6 and 7 show the detailed characteristics of the daily intake of minerals in the diet and the diet with supplements among pregnant women. A significantly higher daily intake of magnesium with the diet among women below 35 years of age compared to older women was found (Table 6). Statistical analysis showed significant differences between a daily intake of iron, zinc and magnesium with a diet and supplements among women taking and not taking supplements (Table 7).

**Table 6:** Intake of minerals in the diet of pregnant women

	Iron (mg/day)	Zinc (mg/day)	Copper (mg/day)	Magnesium (mg/day)	Calcium (mg/day)
Total (*n* = 108)	10.5 ± 2.5	10.5 ± 2.9	1.2 ± 0.3	271.5 ± 77.3	838.5 ± 360.3
< 35 years of age (*n* = 73)	10.7 ± 2.7	10.5 ± 3.1	1.2 ± 0.3	310.0 ± 77.0*	852.1 ± 373.1
≥ 35 years of age (*n* = 33)	10.2 ± 1.9	10.6 ± 2.4	1.2 ± 0.3	295.4 ± 73.1*	824.4 ± 323.4
< 20 week of gestation (*n* = 54)	10.1 ± 2.3	10.1 ± 2.8	1.2 ± 0.3	296.5 ± 69.4	828.3 ± 353.3
≥ 20 week of gestation (*n* = 54)	10.8 ± 2.7	10.9 ± 2.9	1.2 ± 0.3	315.0 ± 80.4	846.4 ± 368.8
Underweight (*n* = 18)	10.6 ± 2.6	10.8 ± 3.1	1.3 ± 0.3	302.9 ± 80.8	866.8 ± 388.5
Normal weight (*n* = 70)	10.6 ± 2.4	10.5 ± 2.8	1.2 ± 0.3	312.9 ± 75.9	835.0 ± 352.9
Overweight (*n* = 12)	9.9 ± 2.5	10.3 ± 3.2	1.1 ± 0.3	272.3 ± 56.4	722.5 ± 337.4
Obese (*n* = 7)	10.1 ± 3.2	10.2 ± 3.2	1.2 ± 0.4	292.0 ± 83.9	934.9 ± 423.0
No mineral supplementation (*n* = 43)	10.9 ± 2.7	10.4 ± 2.7	1.3 ± 0.3	321.3 ± 78.9	857.7 ± 375.1
Mineral supplementation (*n* = 65)	10.1 ± 2.4	10.6 ± .2.9	1.2 ± 0.3	295.5 ± 71.6	823.9 ± 351.3

*p = 0.033

**Table 7:** Intake of minerals in the diet and supplements

	Iron (mg/day)	Zinc (mg/day)	Copper (mg/day)	Magnesium (mg/day)	Calcium (mg/day)
Total (*n* = 108)	30.1 ± 19.1	12.7 ± 3.2	1.6 ± 0.5	326.5 ± 63.1	885.3 ± 343.9
< 35 years of age (*n* = 73)	27.9 ± 19.9	13 ± 3.4	1.7 ± 0.9	376.8 ± 85.4	855.7 ± 371.4
≥ 35 years of age (*n* = 33)	28.2 ± 15.4	12.2 ± 2.7	1.4 ± 0.3	364.9 ± 96.9	827.4 ± 332.7
< 20 week of gestation (*n* = 54)	28.3 ± 12.5	12.4 ± 3.2	1.6 ± 0.9	374.9 ± 81.1	832.3 ± 354.3
≥ 20 week of gestation (*n* = 54)	27.9 ± 13.5	13.3 ± 3.3	1.6 ± 0.7	371.8 ± 95.3	849.3 ± 369.1
Underweight (*n* = 18)	24.2 ± 7.6	13.2 ± 3.5	1.6 ± 0.5	360.9 ± 73.5	871.5 ± 391
Normal weight (*n* = 70)	29.3 ± 14.2	12.7 ± 3.3	1.6 ± 0.9	380.9 ± 92.8	838.3 ± 353.4
Overweight (*n* = 12)	27.9 ± 13.8	12 ± 2.7	1.4 ± 0.4	358.7 ± 87.6	725.3 ± 336.8
Obese (*n* = 7)	26.8 ± 10.4	11.9 ± 2.8	1.4 ± 0.4	351.3 ± 87.7	937.9 ± 422.4
No mineral supplementation (*n* = 43)	11 ± 2.7*	10.4 ± 2.7**	1.3 ± 0.4	321.3 ± 78.9***	857.7 ± 375.1
Mineral supplementation (*n* = 65)	29.2 ± 13.1*	12.7 ± 3.3**	1.5 ± 0.8	367 ± 87.8***	827.3 ± 351.9

*p = 0.016; **p = 0.025; ***p = 0.048

The results of the analysis of the hair showed that in women under 35 years of age, a significantly higher concentration of zinc was found than that in older women (Table 8). Magnesium levels were significantly higher in the hair of women aged 35 years and older than in younger women. Women at or beyond the twentieth week of gestation had significantly lower magnesium concentrations in their hair than women before the twentieth week.

**Table 8:** The content of minerals in hair samples

	Iron (µg/g)	Zinc (µg/g)	Copper (µg/g)	Magnesium (µg/g)	Calcium (µg/g)
Total	23.3 ± 18.8	179.1 ± 50.1	17.3 ± 9.9	87.1 ± 32.4	2311.3 ± 1242.7
< 35 years of age	23.5 ± 20.6	187.3 ± 50.9*	17.8 ± 10.5	78.6 ± 35.5**	2040.4 ± 1236.9
≥ 35 years of age	22.9 ± 13.5	157.1 ± 40.9*	16.2 ± 13.8	95.6 ± 42.5**	2470.2 ± 1256.9
< 20 week of gestation	25.7 ± 23.8	173.5 ± 53.4	17.3 ± 9.7	90.1 ± 41.6***	2320.9 ± 1229.0
≥ 20 week of gestation	20.9 ± 1.9	185.2 ± 46.1	17.3 ± 10.3	74.7 ± 31.4***	1950.9 ± 1256.5
Underweight	21.8 ± 25.6	166.9 ± 35.1	18.9 ± 7.7	80.3 ± 43.3	2128.5 ± 1405.3
Normal weight	24.9 ± 18.7	188.3 ± 47.6	15.8 ± 10.1	81.9 ± 36.3	2186.5 ± 1181.8
Overweight	21.6 ± 11.0	169.7 ± 68.1	25.3 ± 10.3	103.7 ± 36.7	2444.9 ± 1261.1
Obese	16.7 ± 7.2	147.3 ± 53.6	14.2 ± 6.2	69.1 ± 38.8	1677.4 ± 1466.1
No mineral supplementation	20.2 ± 10.9	189.3 ± 47.2	17.9 ± 10.8	96.4 ± 33.9	2542.9 ± 1144.5
Mineral supplementation	25.3 ± 22.0	174.1 ± 52.6	17.3 ± 10.5	88.6 ± 40.0	2281.8 ± 1305.5

*p = 0.042; **p = 0.030; ***p = 0.038

## Discussion

This study has shown that the diet of the pregnant women in the study did not meet the recommended daily energy, carbohydrate and essential micronutrients intake recommendations. There is evidence that a decrease in maternal energy intake impairs mitochondrial function and foetal development [29]. Moreover, it was observed that low maternal carbohydrate intake in the second trimester was related to insufficient total gestational weight gain and neonatal birth weight [30]. The intake of protein and fat in the diet slightly exceeded the recommendations. Similar results were obtained in studies by Gunnarsdottir et al. [31]. It was also observed that the intake of complex carbohydrates was below the recommended level. Only 20% of the recommended amount of fibre was provided in the diet of the pregnant women. Different results were obtained in the study of Liu et al. of pregnant Chinese women, where it was reported that the diets were not balanced in terms of macronutrients [32]. That study also showed a significant excess of fat in the daily diet of the pregnant women.

Our study shows a significant lack of balance in the diet of pregnant women in terms of the intake of micronutrients essential for normal pregnancy (iron, calcium, magnesium, folic acid, and vitamin D). In the studies of Liu et al. and of Zhang et al., the intake of micronutrients was also observed to be significantly low, when compared to the recommendations [32, 33]. In addition, Liu et al. found a significant iron deficiency in the diet of women in the third trimester of pregnancy, when the requirement of the foetus for iron is highest. It should be noted that, during the first 4–6 months of life, the child draws on the iron reserves it accumulated during gestation, mainly in the third trimester [34].

This study has shown that the use of dietary supplements by pregnant women significantly improves the coverage of the recommended intakes of micro-elements. In the case of iron and zinc, supplementation increased the intake above the recommended level. In the studies of de Sá et al. and Nguyen et al., which considered iron supplementation during pregnancy, similar results were found [34, 35]. These authors, however, noted that despite the increase in the intake of iron by supplementation, the hoped-for reduction in the occurrence of anaemia among women was not achieved. In this study, a positive correlation was found between the concentration of iron in the women’s hair and the intake of zinc in the form of dietary supplements. A positive correlation was also demonstrated between the concentration of zinc in the hair and iron supplied with the diet. Knez et al. [36], in line with these results, pointed out that a large proportion of iron deficiency anaemia observed in the populations of many countries may be associated with a simultaneous deficiency of zinc [36]. Bjørklund et al. [37], on the other hand, have drawn attention to the fact that the interaction between zinc and iron depends on nutritional elements, the nutritional status of these micronutrients, and the use of supplements.

In this study, the recommended levels of calcium and magnesium intake were not achieved, despite the use of dietary supplements. Similarly, in the case of vitamin D, supplementation did not allow the recommended daily intake to be reached. Our study also shows that supplementation did not significantly increase the supply of copper and calcium in women. Numerous studies have indicated that the diet of pregnant women is unbalanced in terms of the nutrients essential for bone formation in the foetus [38–40]. Hyde et al. [38] obtained similar values for the calcium and magnesium intake in a study population of pregnant women. Morriset et al. [39] reported higher daily calcium in the diet and, like our study, showed a significant proportion of iron and vitamin D from supplements in the overall intake of these nutrients [39].

This study has shown that the magnesium content in hair samples was significantly higher in older women and significantly lower in women above 20 weeks of gestation. It was found despite no difference in the supply of magnesium with diet and supplements (Table 7). Other authors found that magnesium level in serum during pregnancy was constant [40, 41]. In one of our previous studies, we observed that magnesium concentration in amniotic fluid (AF) decreased as the pregnancy proceeded and we also found a negative correlation between magnesium in AF and foetal growth parameters [42]. We also observed lower magnesium concentration in AF in older women (> 35 years old) than in younger ones [41], however, we did not observe a relation between the age of women and magnesium content in cord blood after delivery [43]. This study shows that the content of zinc in hair samples was significantly lower in older women than in younger (< 35 years old) without significant differences in zinc supply between these groups (Table 7). In other studies of ours, an association between zinc concentration in serum, AF or umbilical cord blood in pregnant women and their age was not found [42, 43]. In opposition to that, Youssof et al. [44] observed significantly higher zinc content in cord blood plasma in older women compared to younger women after delivery.

## Conclusion

The diet of pregnant women aged 18–42 is significantly lacking in balance, especially in the micronutrients crucial to the proper development of the foetus. The use of supplements increases the daily intake of micronutrients. However, in the case of magnesium, calcium, and vitamin D, the recommended intake levels are not achieved. Moreover, the concentration of magnesium and zinc in hair depend on age of the pregnant women and magnesium level in the maternal hair decreases during pregnancy. The low supply of micronutrients in the diet and supplements of pregnant women can have negative health effects for both the woman and the developing foetus.
